# Mechanism insights and experimental feasibility of using boron nitride nanocones for rapid adsorption and degradation of SF_6_ decomposition compounds

**DOI:** 10.1038/s41598-024-78565-2

**Published:** 2024-11-09

**Authors:** Mohammad Hassan Hadizadeh, Yongxia Hu, Fei Xu, Wenxing Wang

**Affiliations:** 1 Environment Research Institute, Shandong University, Qingdao, 266237 China; 2 International Center for Quantum Design of Functional Materials (ICQD), University of Science and Technology of China, Hefei, 230026 China; 3 Shenzhen Research Institute of Shandong University, Shenzhen, 518057 China

**Keywords:** BN nanocones, Adsorption, Degradation, Sulfur-based compounds, DFT-AIMD, Environmental sciences, Chemistry, Nanoscience and technology

## Abstract

Gas-insulated switchgear (GIS) employs sulfur hexafluoride (SF_6_) as an insulating medium to shield electrical gadget. However, SF_6_ can decompose under sure situations, generating dangerous sulfur-based totally compounds which include SO_2_, SOF_2_, and SO_2_F_2_. These byproducts pose enormous dangers to both protection and environmental integrity. Efficiently adsorbing and disposing of those compounds is critical for ensuring operational reliability and reducing environmental dangers. This study investigates the adsorption and degradation mechanisms of SF₆ decomposition compounds (SO₂, SOF₂, and SO₂F₂) on boron nitride nanocones (BNNCs) using density functional theory (DFT) and ab initio molecular dynamics (AIMD) simulations. Our comprehensive analysis covers five distinct systems, exploring individual and combined adsorption scenarios. The findings reveal that the apex of BNNCs plays a crucial role in the adsorption process, showing high efficiency in adsorbing SO₂ (adsorption energy − 1.22 eV) and facilitating the catalytic breakdown of SOF₂ (adsorption energy − 1.57 eV). The positively charged potential at the nanocone’s apex significantly influences the dissociation and subsequent adsorption of fluorine atoms, with an energy barrier for F dissociation at the apex (1.8 kcal/mol) much lower than at the sidewall (5.3 kcal/mol). In gas mixtures, SO₂ preferentially binds to the apex region of BNNCs, with a bond length of approximately 1.38 Å. BNNCs demonstrate superior adsorption capabilities for SO₂ and SOF₂ compared to other boron nitride nanostructures, with adsorption energies up to 89% higher. The electron transfer analysis reveals that BNNC complexes act as potent electron donors, particularly in the case of BNNC@3SO₂F₂. Additionally, BNNCs show significant potential as sensors for detecting SO₂F₂, with a rapid recovery time of 4.67 ps and a notable decrease in the Fermi level energy to -4.97 eV upon adsorption. The study also provides insights into the angular distribution and charge density difference profiles, offering a detailed understanding of the adsorption mechanisms. These findings have important implications for improving the safety and efficiency of gas-insulated switchgear (GIS) and contribute to the development of more effective environmental protection solutions in electrical power systems.

**Supplementary Information:**

The online version contains supplementary material available at 10.1038/s41598-024-78565-2.

## Introduction

Gas-insulated switchgear (GIS) epitomizes the forefront of electrical engineering, encapsulating essential additives within a resilient steel body to reap crucial capabilities which includes separation, switching, transformation, and distribution of electrical power^1^. GIS is pivotal in making sure the reliability of power deliver, interrupting cutting-edge waft, and defensive electrical structures from faults. Its compact and robust design is particularly superb for deployment in city and industrial environments with limited space. A distinctive function of GIS is the usage of sulfur hexafluoride (SF_6_), a medium for its exquisite insulating residences. SF_6_ lets in for a compact and efficient GIS layout because of its high dielectric energy and superior arc-quenching capabilities. However, the operational integrity of GIS may be compromised with the aid of mechanical defects and insulation weaknesses, main to SF_6_ leakage and the formation of dangerous decomposition by-products^2–4^. These by-products, including sulfur dioxide (SO_2_), thionyl fluoride (SOF_2_), and sulfuryl fluoride (SO_2_F_2_), are not only corrosive and toxic but also pose significant risks to equipment reliability and environmental safety^5,6^. This highlights the urgent need for effective methods to eliminate these sulfur-based compounds, ensuring the operational reliability of GIS and mitigating associated environmental and health risks. Recent advancements in gas-insulated switchgear (GIS) technology focus on finding alternatives to SF6, a potent greenhouse gas with a long atmospheric lifetime^7^. Promising alternatives include fluoronitrile and fluoroketone-based mixtures, which have significantly lower global warming potential and are already in use in operational equipment. SF_6_/N_2_ mixtures, particularly with low SF_6_ concentrations (5–30%), show potential for GIS applications^8^. Research efforts have concentrated on evaluating the physical properties, dielectric strengths, and arc-quenching capabilities of these alternative gases^9^. Additionally, improvements in GIS technology include the application of electro-optical technology, high voltage gradient ZnO elements, and new gas circuit breaker structures, contributing to the miniaturization of GIS^8^. The development of SF_6_-free technologies for various applications traditionally using SF_6_ demonstrates the industry’s commitment to reducing environmental impact while maintaining electrical infrastructure reliability^7^. Among the various strategies available for the elimination of sulfur-based compounds, adsorption has emerged as a particularly promising approach. This method stands out over alternatives such as Dielectric Barrier Discharge, Radio Frequency Plasma, and Microwave Plasma due to its lower energy consumption, cost efficiency, and environmental friendliness^10^. Thus, the development of advanced material coatings, is crucial for enhancing the adsorption process.

Nanomaterials exhibit an exceptionally high surface area, which provides more active sites for interactions. This attribute significantly enhances their sensitivity, allowing them to detect even minute quantities of harmful gases with high precision. Furthermore, the rapid response time of these nanomaterials is critical in applications where timely detection and neutralization of toxic substances are essential. This quick responsiveness is attributed to the efficient electron transfer processes and the high mobility of charge carriers within these nanomaterials. Their robust structural integrity under various environmental conditions ensures consistent performance over time, making them reliable for long-term applications. Indeed, the synergy of these properties which includes excessive surface region, greater sensitivity, speedy reaction time, and chemical balance, makes nanomaterials noticeably effective in the detection and neutralization of low concentrations of toxic gases. This capability is especially precious in improving the safety and performance of electronic gadgets, because it allows the development of advanced sensors and protective structures which can perform correctly in diverse and probably hazardous environments.

Nanomaterials can tackle numerous shapes, such as nanotubes, nanosheets, nanowires, and nanocones, each owning awesome properties and applications^11–15^. Recent experimental and theoretical studies have highlighted the significant potential of various nanomaterials in gas sensing and adsorption applications, particularly within the domains of electrical insulation and environmental protection^16,17^. The unique properties of nanomaterials, such as their high surface area, tunable electrical characteristics, and chemical stability, make them ideal candidates for these applications. Boron nitride nanotubes (BNNTs) are particularly noteworthy due to their exceptional thermal stability, electrical insulation properties, and mechanical strength. These characteristics enable BNNTs to be utilized in various applications, including as fillers in composites and as protective coatings, which contribute to enhanced electrical insulation and environmental protection^18–21^. Their ability to resist oxidation and provide thermal management solutions further supports their application in high-performance electronics and environmental remediation technologies^22^. Graphene and its derivatives also play a crucial role in gas sensing applications. The unique electronic properties of graphene allow for high sensitivity in detecting various gases, making it a popular choice for developing advanced sensors^23^. Moreover, the incorporation of graphene into polymer matrices has been shown to enhance the mechanical and electrical properties of the resulting nanocomposites, thereby improving their performance in electrical insulation applications^24^. The use of graphene in combination with other nanomaterials, such as boron nitride, has been explored to create hybrid materials that exhibit both high thermal conductivity and electrical insulation, addressing the challenges of traditional insulating materials^25^. In the context of environmental protection, nanomaterials have been employed for air quality improvement through their application in pollution sensing and remediation. For instance, nanomaterials with large surface areas can effectively adsorb pollutants, thus facilitating cleaner industrial processes and improving ambient air quality^26^. The development of nanocomposites that integrate these materials can lead to innovative solutions for environmental challenges, such as the degradation of hazardous substances and the detection of air pollutants^27^.

Among nanomaterials, nanocones consisting of boron nitride nanocones (BNNCs) show a completely unique and promising opportunity because of their distinct bodily, chemical, and electronic properties. Indeed, the presence of B-N, B-B, and N-N covalent bonds in BNNCs endows them with excessive chemical stability and splendid oxidation resistance, making them suitable for a wide range of packages, inclusive of gas sensors, air purifiers, and protecting coatings^28–31^. Indeed, BNNCs are characterized by superior thermal conductivity and unique structural features that facilitate the high loading efficiency and impressive gas-absorption capabilities, particularly in the context of absorbing toxic gases^28,30–33^. Despite their advantages, the potential of BNNCs for adsorbing SF_6_ decomposition by-products in GIS equipment has not been extensively explored. This is due to several reasons. First, BNNCs are relatively new, and their applications are still under research. Second, testing BNNCs is both expensive and technically challenging. Third, new materials must meet strict industry standards, which can be a lengthy process. Therefore, theoretical work is necessary to reduce the time and cost associated with identifying the benefits of BNNCs for adsorbing SF_6_ decomposition by-products, paving the way for future experimental research. Previous theoretical investigations on gas absorption using BNNCs have primarily relied on density functional theory (DFT) calculations. While DFT is a powerful computational tool that accurately predicts the electronic and structural properties of molecules and materials, it has limitations in studying dynamic processes involving multiple atomic rearrangements over time^34–37^. Conversely, ab initio molecular dynamics (AIMD) is a sophisticated computational technique that merges molecular dynamics simulations with first-principles calculations^38,39^, enabling real-time simulation of molecular and atomic movements^40–43^. In this study, we employ a combination of DFT and AIMD simulations to comprehensively explore the effective reaction sites and pathways during the interaction between sulfur-based compounds and BNNC coatings. Through a range of analyses, including angular and electric charge distribution, time evolution, density of states, electron transfer number, and chemical hardness, we aim to provide a detailed and nuanced understanding of the adsorption process. Our findings illuminate the feasibility and effectiveness of using BNNCs to mitigate SF_6_ decomposition compounds in GIS equipment, blending rigorous theoretical insights with practical applications. This study deepens the understanding of BNNCs’ adsorption mechanisms and their practical applications, significantly contributing to the development of more reliable and environmentally sustainable GIS solutions. Meanwhile, our research underscores the transformative potential of BNNCs in improving electrical safety and mitigating the environmental impact of sulfur-based compounds. This innovative approach not only addresses a critical challenge in the operation of GIS but also paves the way for the broader application of BNNCs in environmental protection and electronic device safety.

## Methods

The examination of the interaction among the disintegration compounds of SF_6_ (SO_2_, SOF_2_, and SO_2_F_2_) and BNNC was carried out by individually situating them in various positions. Molecular dynamics simulations combined to DFT using CP2K’s Quickstep module were executed^44^. The auxiliary plane wave and Gaussian bases alongside the wave functions were used to enhance computational methods by providing greater flexibility and accuracy in simulations^45^. To avoid unwanted interactions, each simulated nanocone was individually placed in a spacious 20 Å cubic container, with a 10 Å vacuum layer above the nanocone. Prior to the AIMD simulations, DFT calculations were performed to optimize each system using the Gaussian software^46^. Subsequently, all systems then underwent a repeated relaxation process using AIMD simulations to ensure they reached their primary equilibrium state. For the AIMD simulations lasting 20 ps, an NVT ensemble was employed, applying a NHC thermostat with a time constant of 0.1 ps^47,48^. The PBE exchange-correlation functional was employed^49^, along with DFT-D3 function without damping^50^. Additionally, the GTH pseudopotentials^51^ were applied in conjunction with the DZVP basis set^52^. The gas molecule adsorption energy, *E*_*a*_, was calculated for different BNNC-gas systems under various conditions based on number of gas molecules. This assessment allowed for a direct comparison of stability and revealed differences in compression energy across different configurations. The equation used to determine *E*_*a*_ is as follows:

1 $$\:{E}_{a}\:=\:{(E}_{\text{B}\text{N}\text{N}\text{C}/\text{g}\text{a}\text{s}}\:\--\:{\text{n}E}_{\text{g}\text{a}\text{s}}\:\--\:{E}_{\text{B}\text{N}\text{N}\text{C}})/n$$

E_BNNC_ signifies the total energy of the nanocone, while *E*_*gas*_ signifies the total energy of sulfur compounds. The adsorption of gas molecules on the BNNC leads to an augmentation of the system’s total energy, denoted as *E*_*BNNC/gas*_. To account for basis set superposition error (BSSE), we applied the counterpoise correction method when calculating the adsorption energies. This correction helps ensure more accurate interaction energies by eliminating artificial stabilization due to basis set incompleteness. The stability of the system was assessed by comparing the resulting energy shift with the initial condition. Where negative *E*_*a*_ indicates an increase in system stability, suggesting a more favorable configuration. Conversely, positive *E*_*a*_ implies a decrease in stability compared to the initial state, indicating a less favorable arrangement. Furthermore, when it comes to the design of materials possessing distinct electronic characteristics, it becomes imperative to grasp the significance of the energy gap (*E*_*g*_). *E*_*g*_ is described as E_LUMO_ - E_HOMO_, signifies the disparity between the highest occupied molecular orbital (HOMO) and the lowest unoccupied molecular orbital (LUMO). Indeed, the *Eg* serves as a quantitative indicator of the amount of energy needed to elevate an electron from the HOMO to the LUMO. Hence, a wider energy gap implies that a greater amount of energy is necessary to stimulate electrons, thereby facilitating the conduction of electricity. A comprehensive density of states (DOS) analysis was conducted on both the pristine BNNC and its complexes. Additionally, the study employed the electron transfer number (∆N) to evaluate the interactions between the adsorbent and various gases. This parameter measures the proportionate amount of electrons that are transferred from the complexes to the corresponding gas molecules of SO_2_, SOF_2_, and SO_2_F_2_, as described below:^53^

2 $$\:\varDelta\:N=\frac{{\mu\:}_{gas}-{\mu\:}_{complex}}{2({{\upeta\:}}_{gas}+{{\upeta\:}}_{complex})}$$

Herein, µ representing the chemical potential and η representing the chemical hardness. When ∆N is negative, the complex assumes the role of an electron donor, facilitating the transfer of charge from the complex to the gas. Conversely, when ∆N is positive, the complex acts as an electron acceptor, enabling the flow of charge from the gas to the complex.

## Results and discussion

### Structure

**Fig. 1:**
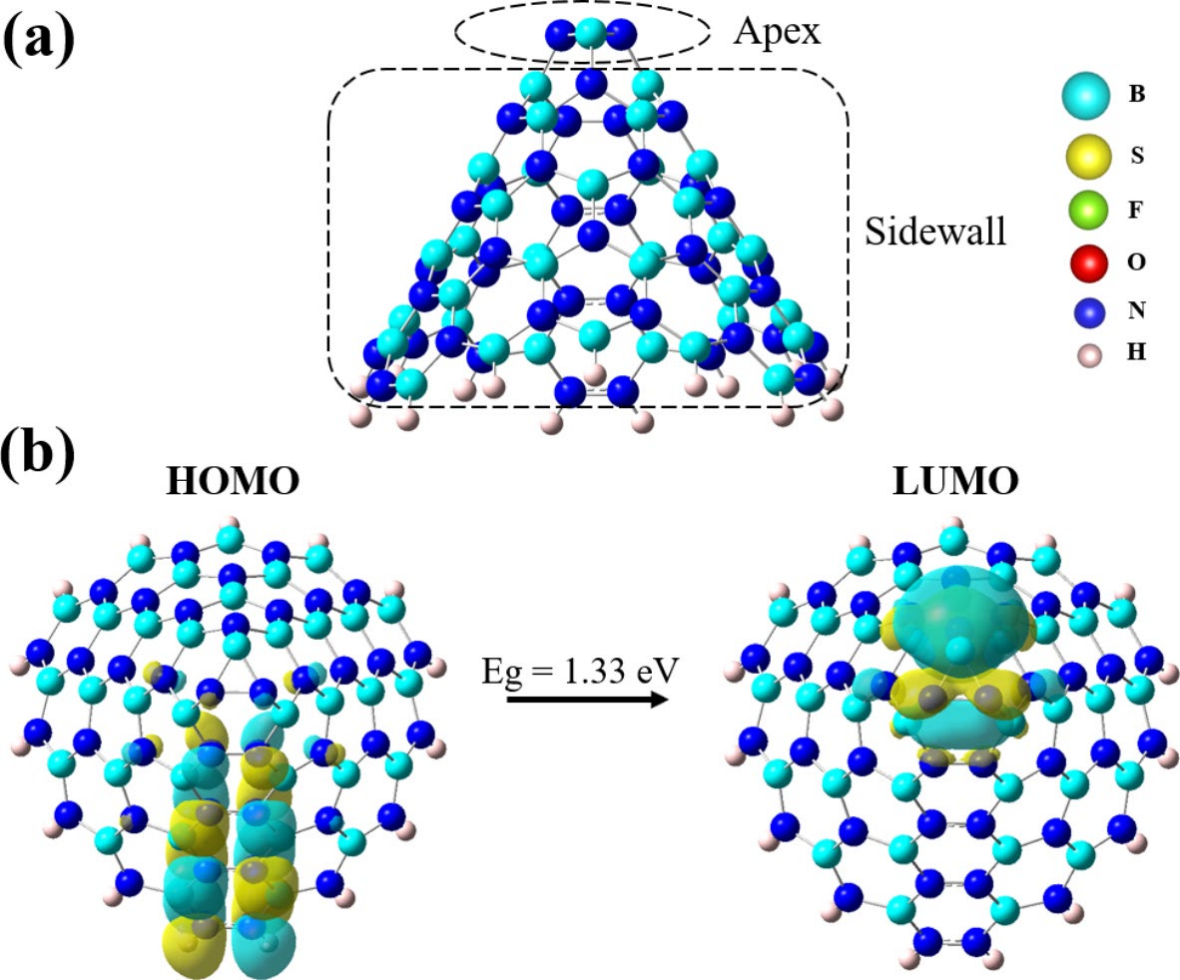
(**a**) A side view of BNNC with a 180° disclination angle. (**b**) The top view of the optimized BNNC structures, showing HOMO and LUMO profiles.

We meticulously selected a specific BNNC characterized by a length of 10 Å and a disclination angle of 180º, motivated by the presence of a triangle apex hole. This particular choice was guided by the anticipated advantages of the BNNC’s distinct structure, offering a vast surface area with a material abundant in defects. Indeed, triangle apex holes in BNNCs can be advantageous as it provides more active sites for interactions with other substances, thereby facilitating enhanced adsorption and reaction mechanisms. This characteristic is beneficial for applications like catalysis and adsorption processes since it exhibits a unique geometry with a B-N-N trigonal ring at its apex (Fig. 1). To initiate our study, we conducted computational simulations employing the Gaussian 16 program^46^, utilizing the B3PW91/6-311G* level. An extensive geometric optimization was conducted on the pristine BNNC, aiming to enhance its structure.

**Fig. 2:**
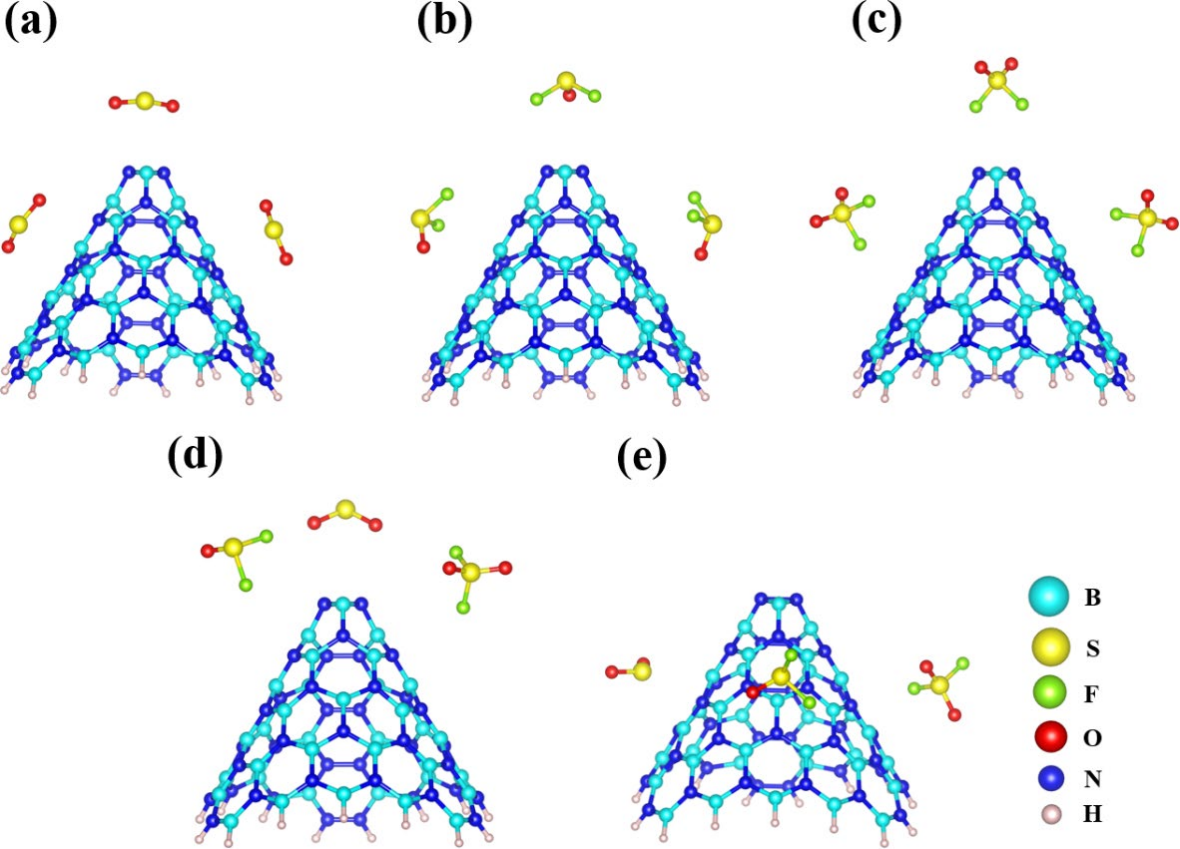
The initial structures used for molecular dynamics calculations: (**a**) BNNC@3SO_2_, (**b**) BNNC@3SOF_2_, (**c**) BNNC@3SO_2_F_2_, (**d**) BNNC@Top, and (**e**) BNNC@Side.

Furthermore, a preliminary assessment of diverse molecular properties was undertaken to acquire valuable insights into the characteristics of BNNC. The calculation of the HOMO/LUMO gap (*E*_*g*_) indicated a value of 1.33 eV, classifying the BNNC as a semiconductor. This finding holds significant implications for various adsorption applications, including gas separation, pollutant removal, environmental monitoring, and purification processes. Furthermore, our analysis showed that the HOMO reveals a distinct distribution surrounding the N-N bonds present on the sidewall of the pristine BNNC. Conversely, the LUMO was primarily observed at the apex of the BNNC, signifying its potential to interact with gas molecules from multiple orientations (Fig. 1). These results highlight the semiconductor nature of the BNNC, as well as its unique electronic structure that facilitates interaction with gas molecules. This knowledge opens up exciting possibilities for utilizing BNNCs in various adsorption-related fields. The inherent charge diversity in gas molecules such as SO_2_, SOF_2_, and SO_2_F_2_ significantly enhances the likelihood of their interaction with various facets of nanocones^54^. Thus, we meticulously positioned the gas molecules in five distinct configurations to probe their interactions with BNNCs, as depicted in Fig. 2. Three systems featured individual 3SO_2_, 3SOF_2_, and 3SO_2_F_2_ molecules, approximately 3.5 Å above and alongside of BNNC, marked with the BNNC@3SO_2_, BNNC@3SOF_2_, and BNNC@3SO_2_F_2_. To investigate the intricacies of competitive adsorption and explore how the simultaneous presence of different gases effects on the adsorption behavior of the BNNC, two additional systems, namely BNNC@Top and BNNC@Side, were also created by arranging three distinct types of gas molecules in a triangular configuration. In the BNNC@Top configuration, all SO_2_, SOF_2_, or SO_2_F_2_ molecules were situated at the apex of the BNNC, while in the BNNC@Side arrangement all gas molecules are positioned along the sidewall of the BNNC. In both models, a uniform spacing of 3.5 Å is maintained between the gas molecules and the surface of the BNNC.

## Exploring SF6 by-products Interaction with BNNC

**Fig. 3:**
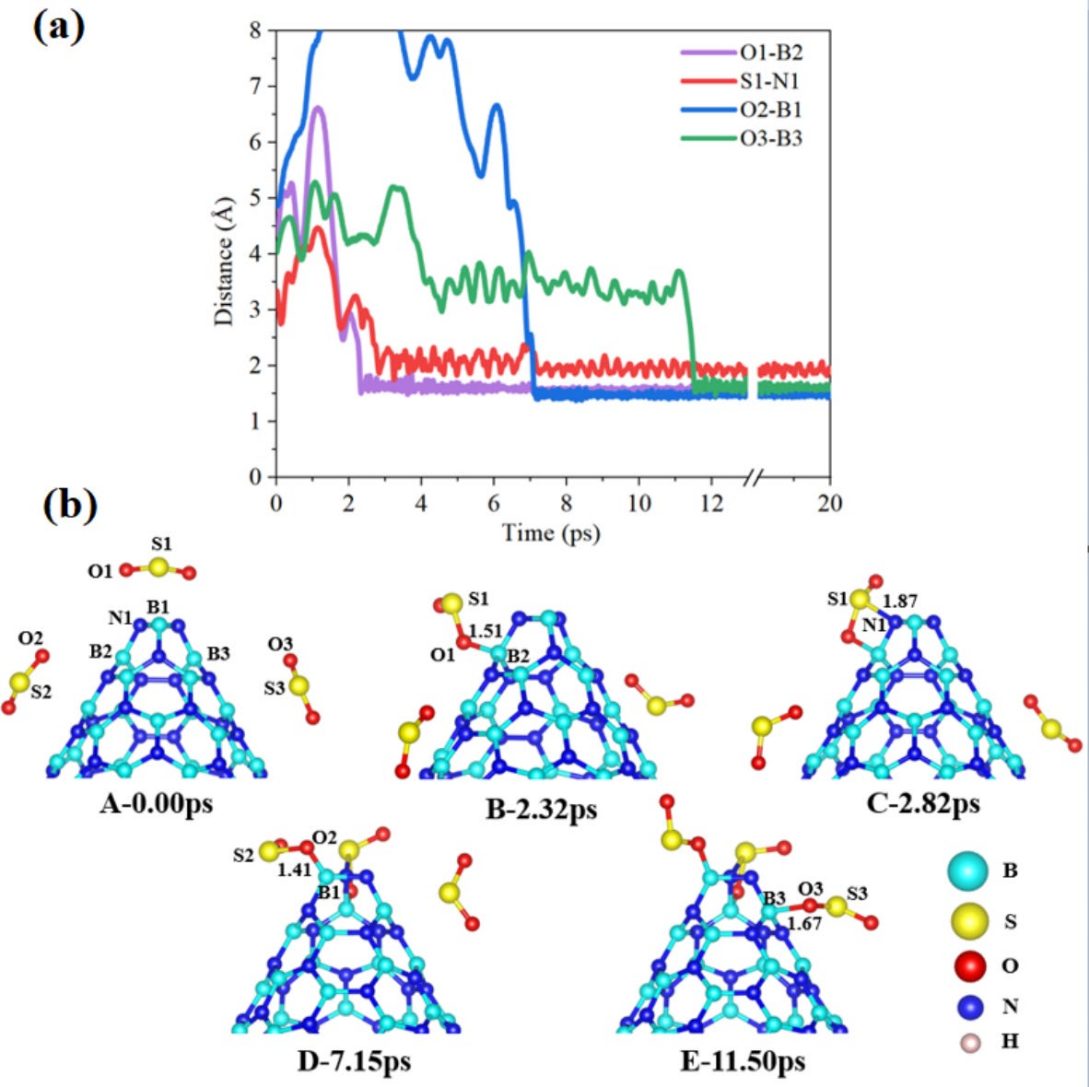
(**a**) A schematic representation and (**b**) time evolution of key bonds during 20 ps of 3SO_2_ gases interacting with BNNC (steps A–E).

In our study, we conducted AIMD calculations to examine the potential degradation and adsorption mechanisms of SO_2_, SOF_2_, and SO_2_F_2_ over a simulation period of 20 ps. During this simulation, we closely analyzed the changes in molecular bonds formed and broken between the gas molecules and BNNNCs over time. Based on the findings illustrated in Fig. 3(a), the initial step involves the approach of one of the SO_2_ molecules located above the cone apex towards B1 and other two SO_2_ molecules located near the sidewall of the BNNC (step A of Fig. 3(b)). The BNNC@3SO_2_ system demonstrated that one of the SO_2_ molecules establishes a bond with boron atom (B2) in the sidewall at a time interval of 2.32 ps. During this particular process, O1 exhibited a connection with B2 at a distance of approximately 1.51 Å (as shown in step B of Fig. 3(b)). Subsequently, at 2.82 ps, S1 formed a bond with N1 located at the cone apex, spanning a distance of approximately 1.87 Å (as depicted in step C of Fig. 3(b)). Following these interactions, another SO_2_ molecule, originating from its oxygen side, bonded with B1 of the cone apex at approximately 1.41 Å after 7.15 ps (step D of Fig. 3(b)). Finally, the third SO_2_ molecule, also from its oxygen side, established a bond with B3 in the sidewall at around 1.67 Å after 11.50 ps (step E of Fig. 3(b)).

**Fig. 4:**
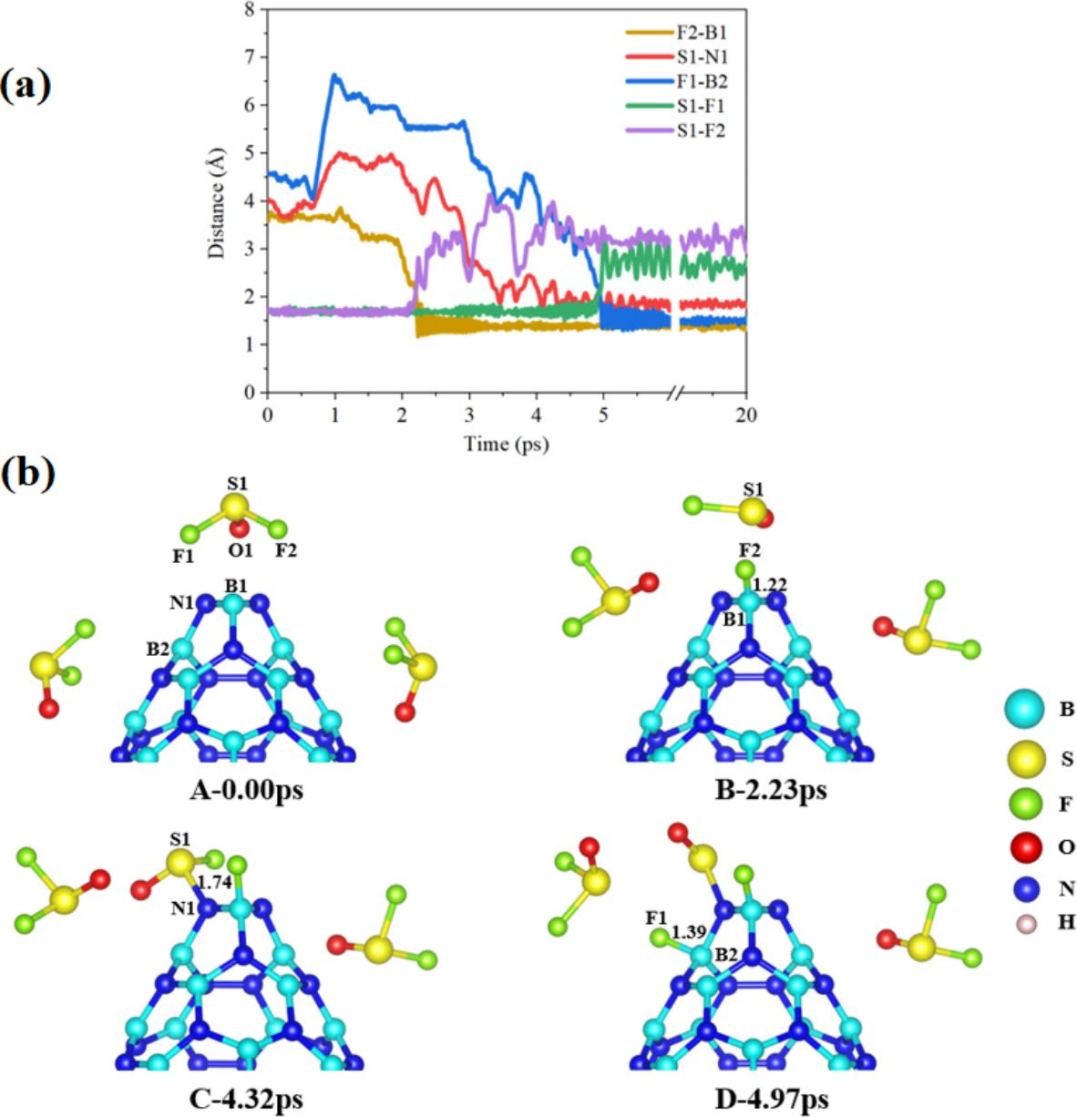
(**a**) A schematic representation and (**b**) time evolution of key bonds during 20 ps of 3SOF_2_ interacting with BNNC (steps A–D).

The main findings of the BNNC@3SO_2_ system reveal that boron atoms within BNNC have a pronounced ability to attract and capture electrons from the oxygen atoms of SO_2_ molecule, a characteristic attributed to the relatively low ionization energy of boron. This electron capture is further facilitated by the polar nature of SO_2_, which possesses lone pairs of electrons on its oxygen atoms. It’s significant to recognize that nitrogen’s greater electronegativity compared to boron results in nitrogen donating electrons, ultimately contributing to the bonding with the sulfur atom of SO_2_ at the cone apex. Overall, the results indicate that the apex of the BNNC demonstrates a higher inclination compared to the sidewall for adsorbing SO_2_ molecules. Meanwhile, As the 3SO_2_ gases interact with BNNC over 20 ps, the consistently shorter O-B bond lengths suggest a stronger bond compared to the S-N bonds during the monitored bond evolution, Fig. 3. In the BNNC@3SOF_2_ system shown in Fig. 4(a), and the initial step involves the approach of one of the SOF_2_ molecules located above the cone apex towards B1 and other two SOF_2_ molecules located near the sidewall of the BNNC (step A of Fig. 4(b)). One observation was made at a time interval of 2.23 ps, indicating that F2 exhibited spatial separation from S1 and displayed an inclination to bind with B1 at the cone apex, at a distance of approximately 1.22 Å (step B of Fig. 4(b)). Subsequently, at 4.32 ps, a connection was established between S1 and N1 at the cone apex, with a bond length of approximately 1.70 Å (step C of Fig. 4(b)). Following this, F1 was observed to move away from S1 and form a connection with B2 at the sidewall, with a bond length of approximately 1.39 Å (step D of Fig. 4(b)). This sequential process ultimately resulted in the detachment of two fluorine atoms from the SOF_2_ molecule located at the cone apex, leading to the adsorption of SO on the cone apex. The two dissociated fluorine atoms were subsequently adsorbed on the cone apex and sidewalls of the BNNC, respectively. Notably, the adsorption process was enhanced by the electrostatic attraction between the positively charged B atoms and the highly electronegative F atoms, which carried a negative charge. Meanwhile, the affinity of the B for capturing F atoms is significantly higher at the apex compared to the sidewall during the interaction of SOF_2_ with BNNC. In contrast, other two SOF_2_ molecules located in the sidewall showed reluctance to interact with the BNNC.

**Fig. 5:**
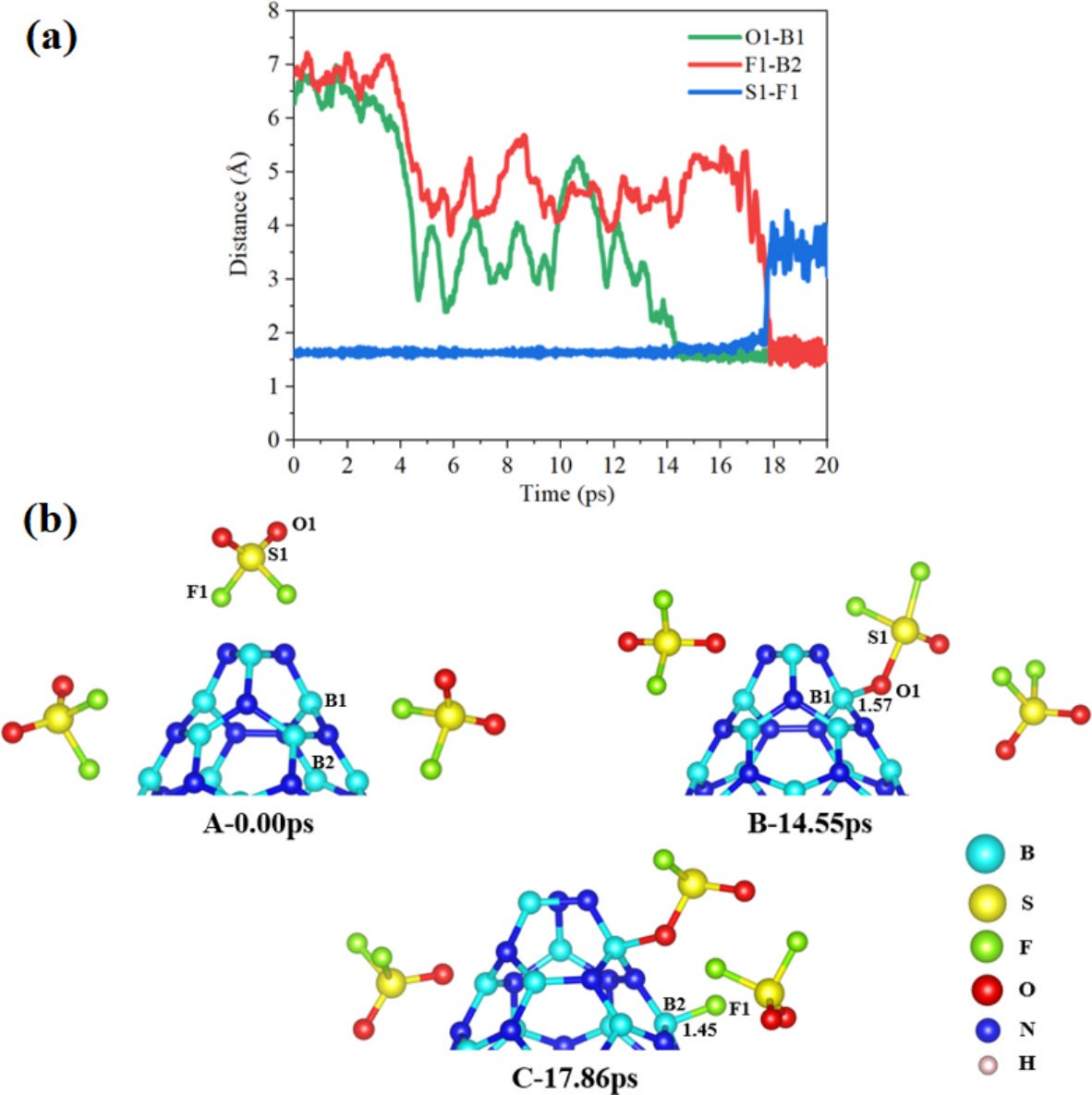
(**a**) A schematic representation and (**b**) time evolution of key bonds during 20 ps of 3SO_2_F_2_ gases interacting with BNNC (steps A–C).

In the BNNC@3SO_2_F_2_ system, the interaction between the BNNC and SO_2_F_2_ molecules occurs in a stepwise manner (Fig. 5 (a)). The initial step involves the approach of one of the SO_2_F_2_ molecules located above the cone apex towards B1 while other two SO_2_F_2_ located on the sidewall of the BNNC (step A of Fig. 5(b)). This approach results in the formation of a chemical bond between the SO_2_F_2_ molecule and the B1 site, with a bond length of approximately 1.57 Å. This bonding event occurs at 14.55 ps, as depicted in step B of Fig. 5(b). Subsequently, a fluorine atom (F1) separates from the SO_2_F_2_ molecule and attaches itself to the B2 site on the sidewall of the BNNC. This process leads to the formation of the SO_2_F structure, where the sulfur atom is bonded to one fluorine atom and two oxygen atoms. This structural transformation occurs at 17.86 ps, as shown in step C of Fig. 5(b). Interestingly, the results indicate that the SO_2_F_2_ molecules located on the sidewall of the BNNC do not exhibit significant interactions with the BNNC itself. Instead, the interactions primarily occur between the SO_2_F_2_ molecule initially positioned above the cone apex of the BNNC. It is noteworthy that in this system, instead of both fluorine atoms being lost in a competitive process, the SO_2_F_2_ molecule tends to release only one fluorine atom during the reaction. This observation suggests a preference for a selective fluorine atom detachment. The underlying factors influencing this selectivity could be attributed to various parameters, such as the stability of the resulting products and the relative energies of the transition states involved in the reaction pathway. Furthermore, an important point to consider is the time scale of the fluorine atom separation in the BNNC@3SO_2_F_2_ system, which occurs around 17.86 ps. This time frame is approximately 15.63 ps longer compared to the separation of first fluorine atom in the BNNC@3SOF_2_ system, which takes place within 2.23 ps. This difference in reaction kinetics suggests that the presence of additional oxygen atoms in the SO_2_F_2_ molecule impacts the overall reaction dynamics and influences the rate of fluorine atom detachment.

**Fig. 6:**
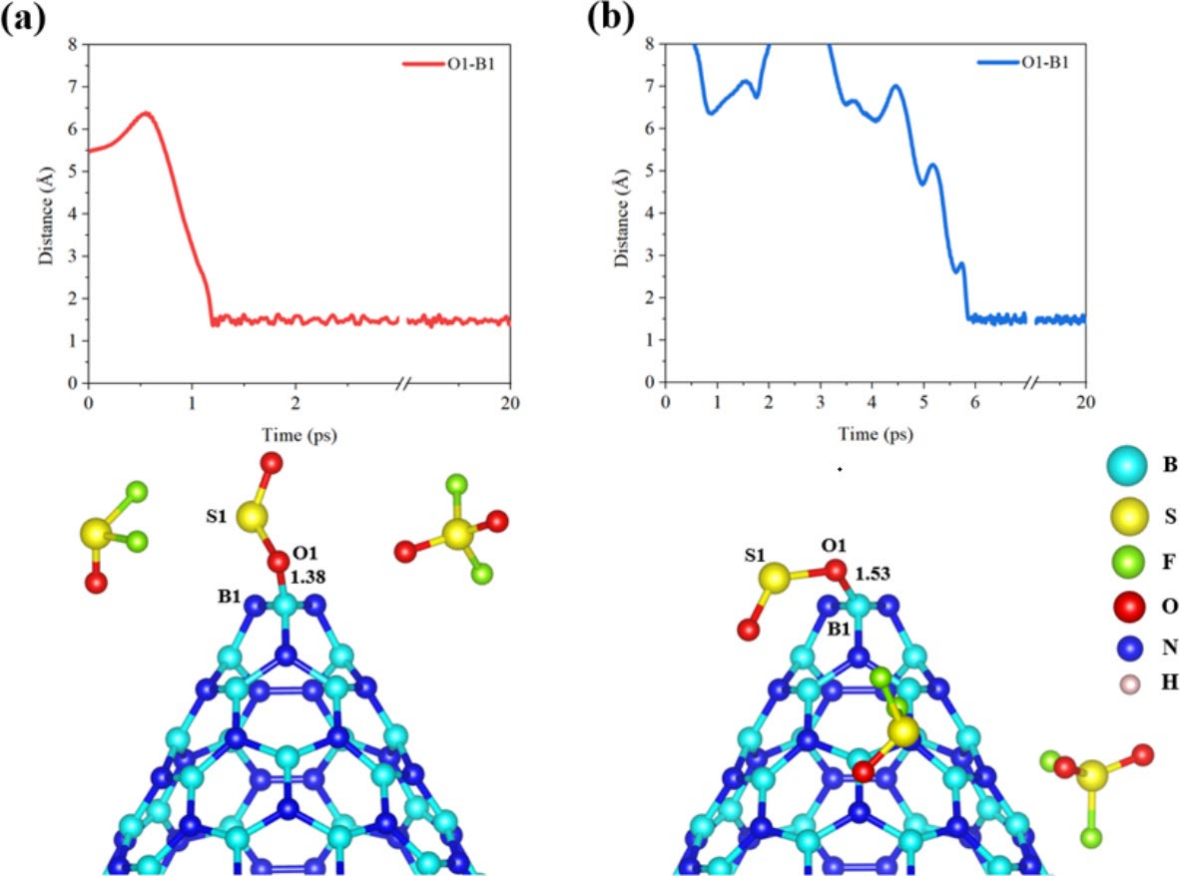
A schematic representation along with time evolution of key bonds during gas interaction with BNNC from (**a**) apex side and (**b**) sidewall.

Generally, the adsorption process in the BNNC@3SOF_2_ (4.97 ps) system occurs approximately 6.53 ps and 12.89 ps earlier than the adsorption processes of the BNNC@3SO_2_ (11.50 ps) and BNNC@3SO_2_F_2_ systems (17.86 ps), respectively. Thus, based on the comparison of reactivity among these compounds, it can be inferred that BNNC@3SOF_2_ displays the highest reactivity, followed by BNNC@3SO_2_, and finally, the BNNC@3SO_2_F_2_ system. The interactions of SO_2_, SOF_2_, and SO_2_F_2_ with BN nanocones are influenced by electronic effects and steric hindrance. SO_2_, without any fluorine atoms, easily forms O-B bonds due to the lack of steric hindrance and the electron-deficient nature of boron. SO_2_F_2_ has fluorines that create steric bulk, but still forms O-B bonds after losing a fluorine atom, suggesting a favorable interaction with boron. SOF_2_, after shedding a fluorine, forms an S-N bond, likely because the loss of fluorine reduces steric hindrance, allowing the sulfur to interact with nitrogen. However, the fully gas adsorption occurred only in BNNC@3SO_2_ systems. It is worth noting that, when SOF_2_ molecule attack from the apex side, it induces the most significant degradation, leading to the separation of both fluorine atoms in the cone apex and sidewall. In the context of the initial positions of the molecules on the sidewall, it is observed that SO_2_ exhibits the ability to be adsorbed onto BNNC from both the apex and sidewall regions. On the other hand, there is no discernible preference for SOF_2_ and SO_2_F_2_ to form bonds with BNNC when they are initially located on the sidewall. Generally, the overall preference for the cone apex in adsorption SO_2_ and degradation SOF_2_ is higher than the sidewall in BNNC systems. The bond lengths between the BNNC and the adsorbent gas molecules in their most stable states are provided in Table S1. Furthermore, in order to examine the adsorption preferences of SO_2_, SOF_2_, and SO_2_F_2_ gases on BNNC, two additional systems were investigated, namely BNNC@Top and BNNC@Side. In the BNNC@Top configuration (Fig. 6(a)), the SO_2_ molecule established a bond of approximately 1.38 Å between its oxygen part (O1) and the boron atom (B1) located at the cone apex at 1.20 ps. Similarly, in the BNNC@Side system (Fig. 6(b)), the SO_2_ molecule formed a bond of approximately 1.53 Å with the boron atom (B1) located at the cone apex through its oxygen moiety (O1) at 5.85 ps. Notice that, no reaction was observed between SOF_2_ or SO_2_F_2_ and BNNC in both systems. The rate at which bonds are formed plays a pivotal role in assessing the reactivity of BNNC@Top and BNNC@Side.

Notably, BNNC@Top exhibits higher reactivity in comparison to BNNC@Side. This discrepancy can be attributed to the initial positioning of the SO_2_ molecule at the apex side, enabling improved accessibility for bonding with the cone apex. Conversely, when the SO_2_ molecule is situated on the side wall, its ability to form bonds is hindered, leading to lower reactivity. It should be emphasized that in both systems, SO_2_ forms a bond exclusively with the boron atom of the cone apex through its oxygen part, albeit with different orientations.

## Analysis of structural and electronic properties

Following a 20 ps AIMD simulation, the most stable complexes of all systems were optimized at the B3PW91/6-311G* level. Our study yielded insightful results regarding the alterations in electronic properties across these systems, as summarized in Table 1. The Fermi level energies (*E*_F_) are fundamental in understanding how electrons behave in materials, influencing both their electrical and thermal characteristics based on their position within the band structure. The adsorption of SO_2_, SOF_2_, and SO_2_F_2_ molecules within the BNNC systems resulted in a significant decrease in the *E*_F_, thereby affecting the electronic structure of the systems. Among the various configurations studied, the BNNC@SO_2_F_2_ configuration exhibited the most significant decrease in E_F_, measuring − 4.97 eV. On the other hand, the BNNC@Side configuration showed the highest E_F_ value after gas adsorption, measuring − 4.61 eV. Overall, the adsorption of gases resulted in a significant change in the E_F_ across all the studied systems, indicating a notable alteration in the electronic properties of the BNNC during adsorption and degradation processes (Figs. 3, 4, 5 and 6).

**Table 1:** The gas molecule adsorption energies (*E*_**a**_**)**, HOMO (*E*_HOMO_), LUMO energies (*E*_LUMO_), Fermi level energies (*E*_F_), energy gap (*E*_g_), chemical hardness (*η*) and electron transfer number (∆*N*) in eV.

Systems	E_a_	E_HOMO_	E_LUMO_	E_F_	E_g_	η	∆*N*
BNNC	–	−4.31	−2.98	−3.64	1.33		
BNNC@3SO_2_	−1.22	−5.10	−4.75	−4.93	0.35	0.18	−59.30
BNNC@3SOF_2_	−1.57	−5.22	−4.66	−4.94	0.55	0.28	−76.14
BNNC@3SO_2_F_2_	−0.22	−5.47	−4.48	−4.97	0.99	0.49	−93.58
BNNC@Top	−0.67	−4.89	−4.38	−4.64	0.50	0.25	−77.80
BNNC@Side	−0.66	−4.85	−4.37	−4.61	0.49	0.24	−77.89

**Fig. 7:**
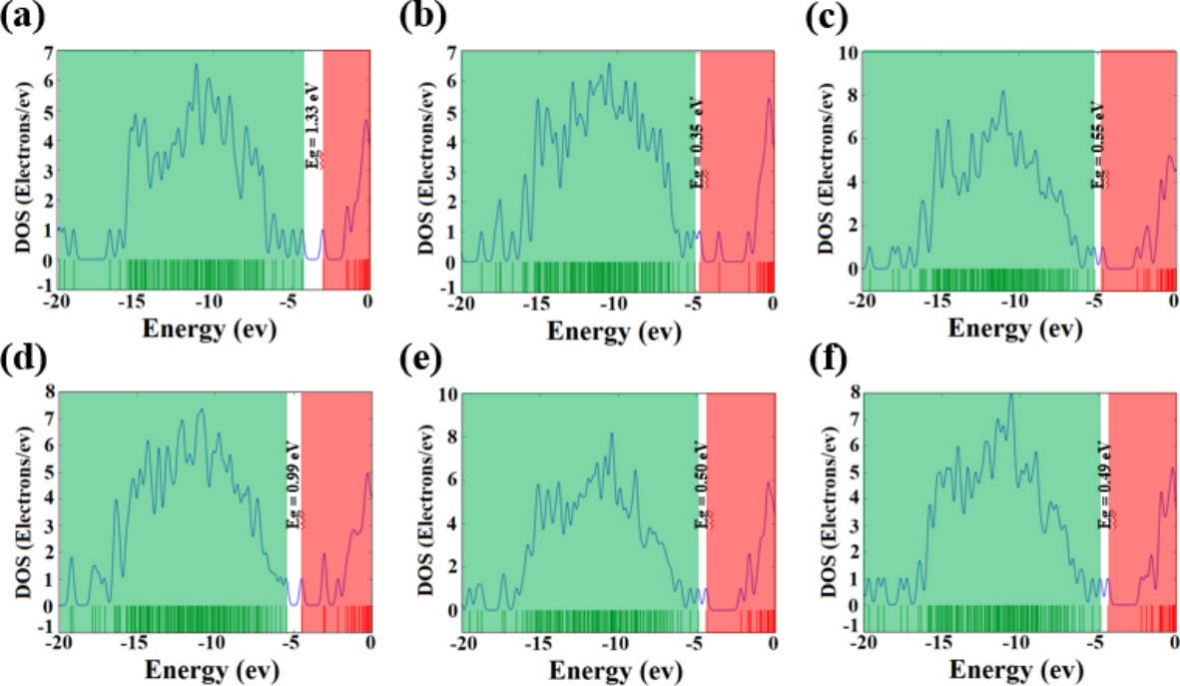
DOS plots for (**a**) pristine BNNC, (**b**) BNNC@3SO_2_, (**c**) BNNC@3SOF_2_, (**d**) BNNC@3SO_2_F_2_, (**e**) BNNC@Top, and (**f**) BNNC@Side systems. The green region and red region correspond to the profiles of HOMO and LUMO, respectively.

Meanwhile, the evaluation of chemical hardness (*η*) is vital for assessing a molecule’s stability, as it quantifies its resistance to electron transfer. Remarkably, among the different systems, the BNNC@SO_2_F_2_ system demonstrated the highest η value, suggesting that it has the least tendency to engage in additional chemical reactions with other gas molecules. Conversely, when the interaction occurred between BNNC and the gas molecules from the sidewall (BNNC@Side), the system displayed the lowest resistance to electron transfer. These results indicate that the BNNC@SO_2_F_2_ system is less prone to undergo further reactions, while the BNNC@Side system is more inclined to engage in additional electron transfer and chemical reactions. Investigations into gas molecule adsorption energies (E_a_) indicate that the approach of SOF_2_ to the apex of BNNC (BNNC@3SOF_2_) achieves the most favorable energy level, with a relative energy of −1.57 eV. On the other hand, the adsorption of SO_2_F_2_ gases onto BNNC (BNNC@3SO_2_F_2_) shows the least favorable energy at -0.22 eV. Thus, the adsorption energy ranking, from most to least favorable, in the presence of three identical molecules is as follows: BNNC@3SOF_2_ exhibits the strongest adsorption energy, followed by BNNC@3SO_2_, and BNNC@3SO_2_F_2_ displays the least favorable adsorption energy. Noted that the adsorption energy shows no substantial variation when examining the adsorption of all gases in both the BNNC@Top and BNNC@Side configurations. This result, no substantial variation, is in accordance with the observation that during the adsorption process in both BNNC@Top and BNNC@Side systems, only SO_2_ from the apex cone demonstrates a significant bonding interaction with BNNC. Upon examining Fig. 7, which illustrates the density of states (DOS), it becomes evident that the adsorption of gases onto BNNC structures leads to a noteworthy effect on their electronic properties. Specifically, the energy difference between the HOMO and LUMO, which defines the bandgap energy (*E*_*g*_), is reduced after gas molecules adhere to the nanocones. The DOS plots clearly demonstrate that this reduction in *E*_*g*_ is a general consequence of gas adsorption, with SO_2_ exerting the most significant influence. The interaction between SO_2_ and the nanocone material appears to alter the distribution of electronic states more drastically than other gases, potentially affecting the material’s electrical and optical behavior in a manner that could be beneficial for applications in electronic and sensing devices. Furthermore, in order to deepen our insight into the process of gas release from BNNC structures, we calculated the time needed for the system to revert to its original condition, referred to as the recovery time (τ), by employing the subsequent equation:

3 $$\:\tau\:={{\upnu\:}}^{-1}\text{e}\text{x}\text{p}(-E/kT)$$

Here, ν represents the frequency of visible light, T denotes the temperature (300 K), and k represents Boltzmann’s constant (approximately 1.99 kcal mol^−1^K^−1^). The results of the recovery time related to SF_6_ decomposition by-products showed that BNNC@SO_2_F_2_ exhibits the minimum value of τ (4.67ps) among all systems. Thus, taking into account the adsorption energy of −0.22 in BNNC@SO_2_F_2_ and the simultaneous decrease in E_g_, we can anticipate that BNNC has the potential to function as an exceptional sensor for SO_2_F_2_ gases. As a comparison, while the PdPTe monolayer shows promise for SO_2_ detection with a 4.42 s recovery time at 498 K, the BNNC system demonstrates superior performance overall^55^. Indeed, BNNC exhibits an exceptionally short recovery time of 4.67 ps for SO_2_F_2_, significantly faster than PdPTe. BNNCs also show higher adsorption energies for SO_2_ and SOF_2_, up to 89% higher than other boron nitride structures. The BNNC system’s versatility in detecting multiple gases and ability to catalytically break down SOF_2_ make it a more efficient and versatile choice for sensing and potentially decomposing SF_6_ breakdown products in gas-insulated switchgear applications.

As a comparison with AlNNC for similar applications^56^, BNNCs excel in apex-centered adsorption, especially for SO_2_ and SOF_2_, with high SO_2_F_2_ sensing potential. They show superior adsorption for SO_2_ and SOF_2_ compared to other BN structures, with a reactivity order of BNNC@3SOF_2_ > BNNC@3SO_2_ > BNNC@3SO_2_F_2_ and faster recovery time. While AlNNCs favor sidewall adsorption/degradation, BNNCs offer unique advantages in apex-centered mechanisms, though AlNNCs may be better for multi-gas adsorption. The choice depends on specific application needs. In order to thoroughly assess gas absorption in various BN nanostructures, we performed a comparative study by referencing pertinent research findings^57–61^. As shown in Table 2, the findings highlight the our BNNC structure’s remarkable ability to adsorb SO_2_ and SOF_2_, surpassing other BN nanostructural forms. Indeed, the distinctive surface properties of the nanocone provide an abundance of active sites, significantly enhancing its adsorption capacity for these molecules. However, its capacity for absorbing SO_2_F_2_ is not as effective as most other BN nanostructures. Thus, it can be inferred that BNNC exhibits a strong selective absorption or reaction towards SOF_2_ and SO_2_, rather than SO_2_F_2_. It should be noted that previous studies have not investigated the simultaneous adsorption and degradation mechanisms of SO_2_, SOF_2_, and SO_2_F_2_. These simultaneous adsorption mechanisms are important to be considered because they can influence the interactions between these gases and the material’s structure during concurrent adsorption.

**Table 2:** The gas molecule adsorption energies (*E*_*a*_) related to various BN nanostructures.

Type	Energy (eV)			Ref
	SO_2_	SOF_2_	SO_2_F_2_	
Nanocone	−1.22	−1.57	−0.22	Our result
Pd-monolayer	−0.83	−0.94	−0.48	^57^
P-monolayer	–	−0.16	−0.36	^58^
F-BN-H	−0.70	−0.40	−0.25	^59^
Ga@nanotube	–	−0.99	−0.86	^60^
Ni@nanotube	−0.86	−0.52	−0.22	^61^

* The methodology consistently employed across various references involves the utilization of the DFT with the PBE exchange-correlation functional.

**Fig. 8:**
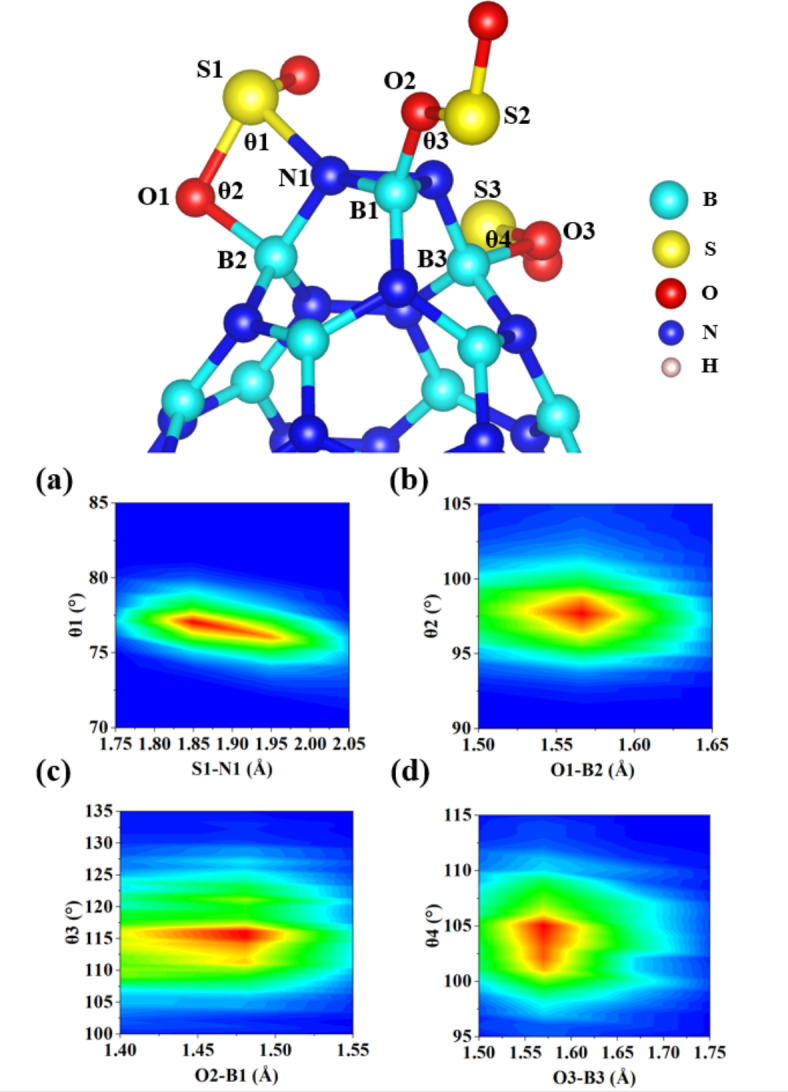
Angular analysis related to the dominant structure of BNNC@3SO_2_.

The analysis of electron transfer number (∆N) has yielded intriguing insights into the behavior of BNNC complexes in terms of their capacity to donate electrons to other gases, as presented in Table 1. The findings unambiguously indicate that each of the BNNC complexes shows a significant tendency to act as potent electron donors, as evidenced by a negative electron transfer number (∆*N* < 0), particularly in the case of BNNC@3SO_2_F_2_. This electron-donating ability of BNNC enables its active involvement in redox reactions with diverse gases, thus offering potential for the development of efficient gas purification systems and pollutant removal technologies. In addition, the size and configuration of HOMO orbitals are critical for understanding the electron-donating capabilities of these systems. As a general rule, a more substantial HOMO suggests a heightened electron-donating potential due to the increased electron delocalization over the molecule. Initial studies point out that the HOMO orbitals are usually shaped by interactions with the sidewalls, where nitrogen atoms are often bonded together (N-N), as shown in Fig. S1. This confirm that N-based functional groups are key in defining the electronic structure and reactivity of diverse systems. Moreover, studying angular distribution analysis can enhance spectroscopic methods like X-ray diffraction and neutron scattering by helping determine molecular structures and revealing the two-dimensional spatial arrangement. This analytical approach plays a pivotal role in comprehending the conformational stability and reactivity of systems involving BNNC complexes. Indeed, by calculating the two-dimensional distribution functions P(θ, r), valuable information concerning the structural properties and stability of various chemical systems can be obtained. In the present study, the outcomes of P(θ, r) for the most favorable system based on multiple adsorption (BNNC@3SO_2_) revealed that the second SO_2_ molecule forms the strongest bond at an angle of approximately 115^º^ and a bond length of 1.47 Å. It is worth mentioning that the first and third SO_2_ molecules exhibit identical bond lengths with BNNC (O1-B2, O3-B3 ≈ 1.56 Å), but with different orientations 97^º^ and 103^º^, respectively (refer to Fig. 8).

**Fig. 9:**
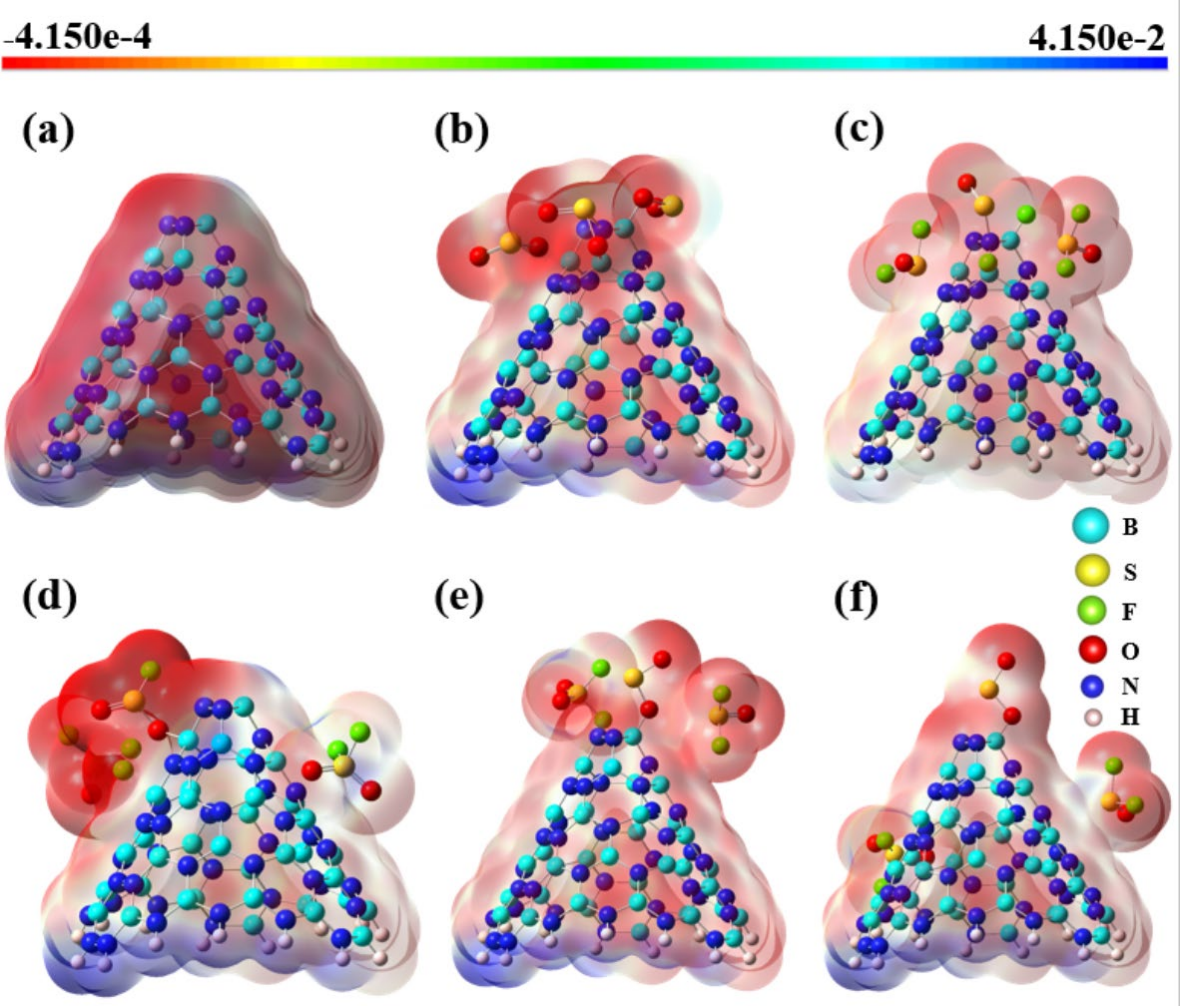
Molecular electrostatic potential maps of (**a**) pristine BNNC, (**b**) BNNC@3SO_2_, (**c**) BNNC@3SOF_2_, (**d**) BNNC@3SO_2_F_2_, (**e**) BNNC@Top, and (**f**) BNNC@Side.

These findings indicate that the strongest adsorption in BNNC@3SO_2_ occurs when the oxygen portion of the SO_2_ molecule establishes a connection with the boron atom located at the apex of the cone-shaped BNNC structure, with minimal influence from other molecules. The angular distribution patterns for the BNNC@3SOF_2_, BNNC@3SO_2_F_2_, BNNC@Top, and BNNC@Side systems are depicted in Fig. S2. From other side, the molecular electrostatic potential surface (EPS) offers a visual map of charge distribution, guiding us through the complex landscape of adsorption by pinpointing reactive sites and illustrating the orientation and strength of molecular interactions. In this study, we employed EPS visualization to analyze the electric charge distribution within BNNC complexes, as depicted in Fig. 9. By examining the EPS of both the pristine BNNC and its complexes, we observed distinct regions characterized by high electron density, indicated by the red color, as well as regions with lower electron density, represented by the blue color. The apex and sidewall of pristine BNNC is characterized by a pronounced accumulation of negative charge, predominantly observed in the N-N bonds. Consequently, it can be inferred that SF_6_ decomposition by-products exhibit a selective affinity for this particular region, with a notable preference for their S component. This statement is in complete accordance with schematical representation in Figs. 3 and 4. Conversely, the B atoms in the apex and sidewall exhibit a considerably higher concentration of positive charge. This suggests that SF_6_ decomposition by-products tend to adsorb onto the B atom of pristine BNNCs, specifically through their O portion. Furthermore, the positively charged potential present on the cone apex and sidewall of the nanocone serves as the primary driving force behind the dissociation and subsequent adsorption of fluorine (F) atoms. Indeed, the positive potential facilitates interactions with negatively charged species, thereby promoting the adsorption process. This statement impeccably corresponds to the meticulously illustrated schematics showcased in Figs. 3, 4, 5 and 6. Notably, the adsorption in the BNNC@3SO_2_ and BNNC@3SO_2_F_2_ systems lead to the highest electron density among all systems, thereby rendering it more susceptible to reactions with other positively charged species. The atomic partial charges, as well as the bond lengths of the most stable structures, are visually presented in Fig. S3.

**Fig. 10:**
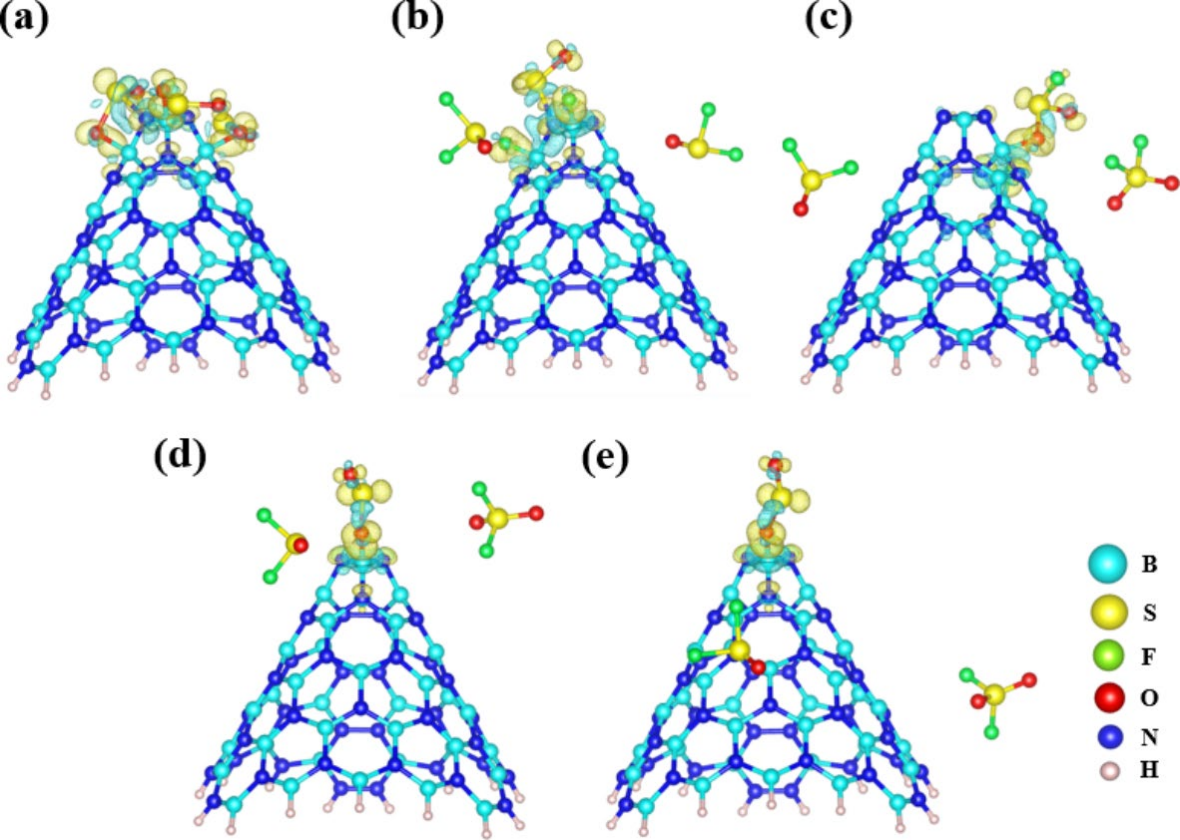
Charge density difference for (**a**) BNNC@3SO_2_, (**b**) BNNC@3SOF_2_, (**c**) BNNC@3SO_2_F_2_, (**d**) BNNC@Top, and (**e**) BNNC@Side.

The role of Charge Density Difference (CDD) is foundational in dissecting the nuances of gas adsorption, as it illuminates the subtleties of charge redistribution among gas molecules and adsorbents. Our research leveraged CDD as a tool to delve into the charge transfer interactions between the gases SO_2_, SOF_2_, SO_2_F_2_, and BNNCs. Depicted in Fig. 10 are the CDD profiles for the most energetically favorable configurations, including BNNC complexes with individual SO_2_, SOF_2_, SO_2_F_2_, where interactions occurring at the top and side sites of BNNC. To quantify the CDD, we applied the equation Δρ = ρ(complex) - [ρ(BNNC) + ρ(gas)], where ρ(complex) represents the charge density of the gas adsorbed on the BNNC, ρ(BNNC) is the charge density of the standalone BNNC, and ρ(gas) corresponds to the charge density of the gas molecules. In the graphical representation of Fig. 10, charge accumulations are particularly noticeable on the adsorbed atoms of S, O, and F in the complexes. Where, the BNNC@3SO_2_ configuration demonstrates significant electron charge concentration, as evidenced by the yellow areas at the cone apex. These zones of increased electron density align with the data presented in Table 1, showing these structures to possess greater adsorption capabilities.

## Experimental feasibility

**Fig. 11:**
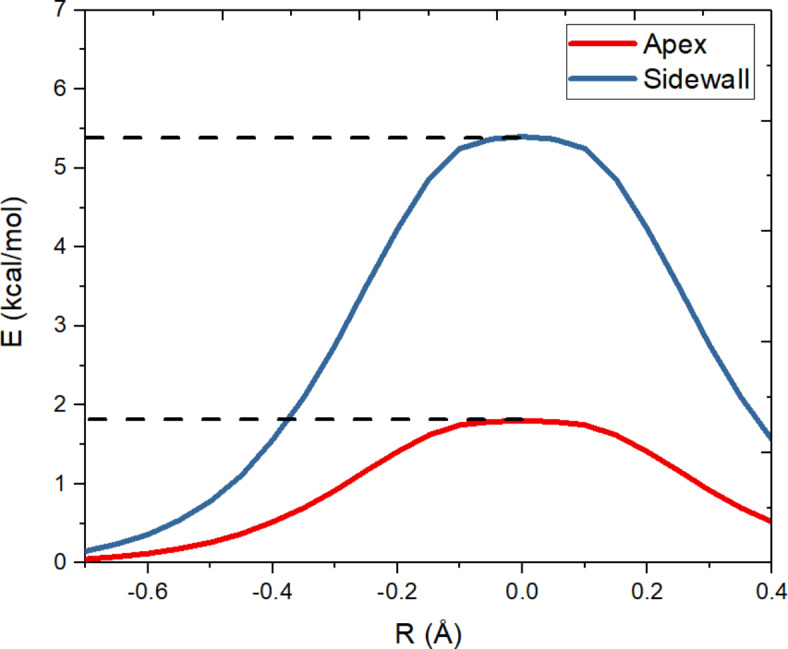
The energy barrier for dissociation of F from SOF_2_ from apex cone and sidewall of BNNC.

The findings of this study have substantial implications for the experimental feasibility of using BNNCs to manage SF_6_ decomposition by-products in gas-insulated switchgear. By employing comprehensive density functional theory and ab initio molecular dynamics simulations, we have elucidated the adsorption mechanisms of hazardous sulfur compounds such as SO_2_, SOF_2_, and SO_2_F_2_ on BNNCs. These theoretical insights provide a robust foundation for experimental endeavors, significantly reducing the time and cost associated with empirical trials. The apex of the BNNCs has been identified as a critical site for adsorption, showing high efficiency in adsorbing SO₂ and facilitating the catalytic breakdown of SOF_2_. This is attributed to the positively charged potential at the nanocone’s apex, which influences the dissociation and adsorption of fluorine atoms. We used constrained molecular dynamics to compare the energy barrier during the dissociation of F from SOF_2_, focusing on both the apex cone and the side wall, Fig. 11, shown that energy barrier for breaking F at the apex cone (1.8 kcal/mol) is much lower than sidewall (5.3 kcal/mole). This disparity suggests that the positively charged potential at the apex facilitates the dissociation process, thereby enhancing catalytic activity. Furthermore, in gas mixtures, SO_2_ preferentially binds to the apex region of BNNCs, underscoring the specificity and efficiency of BNNCs in adsorbing particular sulfur compounds. This specificity could be leveraged in experimental settings to design BNNC-based sensors and adsorbents with enhanced performance metrics. Experimental feasibility is further supported by the significant potential of BNNCs as sensors for detecting SO₂F₂, outperforming other boron nitride nanostructures in adsorption efficiency. This research not only highlights the theoretical underpinnings of BNNCs’ adsorption capabilities but also paves the way for practical applications in environmental monitoring and protection. The robust structural integrity, high surface area, and unique electronic properties of BNNCs facilitate rapid and precise detection of toxic gases, making them ideal for long-term use in various environmental conditions. The experimental implementation of BNNCs can be expedited by our detailed understanding of their interaction mechanisms with sulfur-based compounds. These insights enable the design of targeted experiments to validate theoretical predictions, optimize material properties, and develop scalable production methods. Additionally, the combination of DFT and AIMD simulations offers a dynamic perspective on the adsorption process, allowing researchers to anticipate and mitigate potential challenges in real-time applications. Indeed, the synergy between theoretical insights and experimental feasibility demonstrated in this study underscores the transformative potential of BNNCs in enhancing the safety and reliability of GIS. By advancing our understanding of BNNCs’ adsorption mechanisms, this research contributes to the development of more effective and environmentally friendly solutions for managing SF_6_ decomposition by-products, ultimately benefiting both the power systems industry and environmental conservation efforts. Moreover, the integration of BNNCs in GIS presents an opportunity to significantly enhance the operational lifespan and efficiency of these systems. The high reactivity and selective adsorption properties of BNNCs not only improve the purification process but also minimize the impact of corrosive by-products on system components. This could lead to a reduction in maintenance costs and downtime, offering substantial economic benefits. Additionally, the eco-friendly nature of BNNCs aligns with global efforts to reduce the environmental footprint of industrial technologies. As regulatory pressures increase to minimize greenhouse gas emissions and mitigate climate change, the adoption of BNNCs could serve as a pivotal strategy in achieving these goals. The potential for BNNCs to be engineered at the nanoscale allows for precise customization of their properties, making them adaptable to various industrial requirements. This flexibility extends to the potential development of hybrid materials, combining BNNCs with other nanostructures to further enhance their functional capabilities. The ongoing research into BNNCs also opens avenues for interdisciplinary collaboration, integrating materials science, environmental engineering, and computational chemistry to drive innovation. As such, the findings from this study not only contribute to the scientific community but also have the potential to influence policy-making and industrial practices on a global scale.

## Conclusion

This comprehensive study provides novel insights into the interactions between SO₂, SOF₂, and SO₂F₂ with BNNC structures using DFT and AIMD simulations. Our detailed analysis of five systems, covering all orientations during individual and combined adsorption, has led to a deep understanding of bond dynamics and reaction mechanisms. The reactivity sequence for individual gas molecules interacting with pristine BNNC is BNNC@3SOF₂ > BNNC@3SO₂ > BNNC@3SO₂F₂, with the highest adsorption energy of -1.57 eV observed for SOF₂ adsorbed on the BNNC from the cone apex while undergoing simultaneous F degradation. In the BNNC@3SO₂ system, SO₂ molecules show a greater tendency to undergo adsorption at the apex of the cone structure, with bond lengths ranging from 1.47 Å to 1.56 Å and angular distributions between 97° and 115°. In both the BNNC@3SOF₂ and BNNC@3SO₂F₂ systems, molecules initially located at the apex experience degradation, with F atoms dissociating and forming bonds with B atoms. The BNNC@Top configuration demonstrates higher reactivity than BNNC@Side due to the initial positioning of the SO₂ molecule at the apex side, attributed to the higher electron density at the N-N-B triangular hole at the cone apex. This is further supported by the molecular electrostatic potential surface analysis, which reveals distinct regions of high electron density at the apex and sidewall of pristine BNNC. Our findings conclusively demonstrate that BNNC exhibits superior adsorption of SO₂ and SOF₂ compared to other BN nanostructures, with adsorption energies up to 89% higher. The electron transfer number (ΔN) analysis indicates that each of the BNNC complexes shows a significant tendency to act as potent electron donors, particularly in the case of BNNC@3SO₂F₂. This electron-donating ability enables BNNC’s active involvement in redox reactions with diverse gases, offering potential for the development of efficient gas purification systems and pollutant removal technologies. Furthermore, BNNCs show exceptional sensing capabilities for SO₂F₂ gases, with a rapid recovery time of 4.67 ps and a notable decrease in the Fermi level energy to -4.97 eV upon adsorption. The charge density difference analysis reveals significant electron charge concentration at the cone apex, particularly in the BNNC@3SO₂ configuration, aligning with the observed higher adsorption capabilities. These results have significant implications for the design of BNNC-based sensors and adsorbents for GIS applications. The high reactivity and selective adsorption properties of BNNCs not only improve the purification process but also minimize the impact of corrosive by-products on system components. This could lead to a reduction in maintenance costs and downtime, offering substantial economic benefits. Additionally, the eco-friendly nature of BNNCs aligns with global efforts to reduce the environmental footprint of industrial technologies. Future research should focus on experimental validation of these theoretical findings and optimization of BNNC structures for specific gas sensing and adsorption applications. This could include investigating the effects of doping or functionalization on BNNC performance, as well as exploring the potential of hybrid materials combining BNNCs with other nanostructures. While our study provides valuable insights, it is important to consider potential challenges in scaling up BNNC production for practical applications. Further research is needed to develop cost-effective and scalable synthesis methods for BNNCs with controlled size and morphology. The integration of BNNCs in GIS technology could lead to substantial improvements in operational reliability, safety, and environmental protection. As regulatory pressures increase to minimize greenhouse gas emissions and mitigate climate change, the adoption of BNNCs could serve as a pivotal strategy in achieving these goals. This research not only advances our understanding of nanomaterial-gas interactions but also paves the way for the development of more efficient and environmentally friendly solutions for managing SF₆ decomposition byproducts in electrical power systems. The potential impact of this work extends beyond the immediate field of GIS technology, offering insights that could be applied to broader areas of environmental remediation, gas sensing, and nanomaterial-based technologies.

## Electronic supplementary material

Below is the link to the electronic supplementary material.

Supplementary Material 1

## Data Availability

All data generated or analysed during this study are included in this published article.
