# Supplementary material for: Mechanism insights and experimental feasibility of using boron nitride nanocones for rapid adsorption and degradation of SF6 decomposition compounds
Source: Sci Rep. 2024 Nov 9;14:27317. doi: 10.1038/s41598-024-78565-2 (PMC11549217; doi:10.1038/s41598-024-78565-2)
Supplement: Supplementary file 1 — Supplementary Material 1 [file 41598_2024_78565_MOESM1_ESM.docx]

**Supporting Information**

**Mechanism Insights and Experimental Feasibility of Using Boron Nitride Nanocones for Rapid Adsorption and Degradation of SF_6_ Decomposition Compounds**

Mohammad Hassan Hadizadeh^1,2^, Yongxia Hu^1^, Fei Xu^1,3*^, Wenxing Wang^1^

*^1^Environment Research Institute, Shandong University, Qingdao 266237, China*

*^2^International Center for Quantum Design of Functional Materials (ICQD), University of Science and Technology of China, Hefei 230026, China*

*^3^Shenzhen Research Institute of Shandong University, Shenzhen 518057, China*


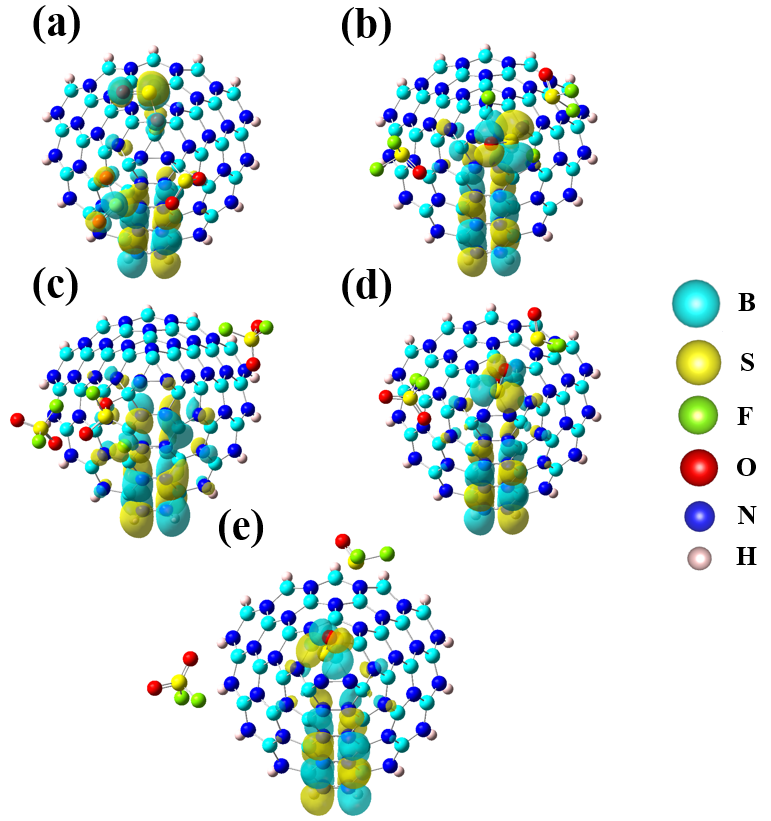


**Figure S1.** HOMO profiles related to dominant structure of (a) BNNC@3SO_2_, (b) BNNC@3SOF_2_, (c) BNNC@3SO_2_F_2_, (d) BNNC@Top, (e) BNNC@Side systems.


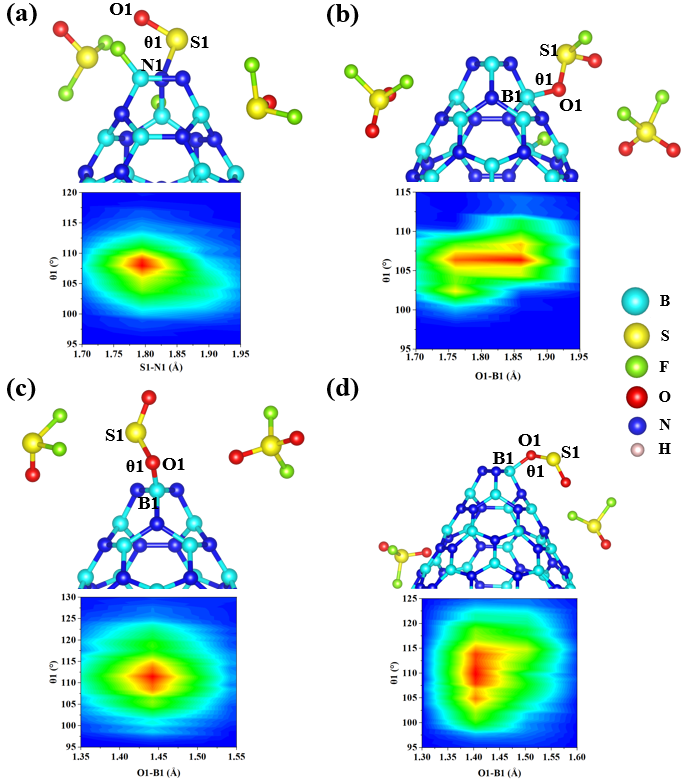


**Figure S2.** Angular analysis related to dominant structure of (a) BNNC@3SOF_2_, (b) BNNC@3SO_2_F_2_, (c) BNNC@Top, and (d) BNNC@Side systems


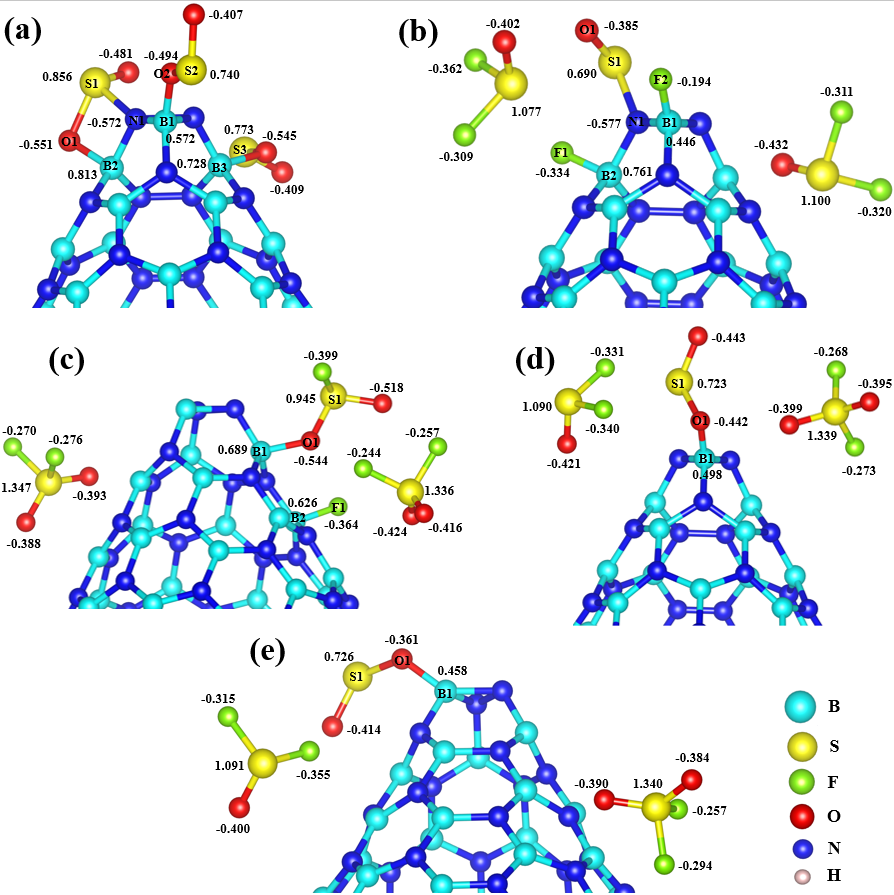


**Figure S3**. The atomic partial charges related to the most stable structures of (a) BNNC@3SO_2_, (b) BNNC@3SOF_2_, (c) BNNC@3SO_2_F_2_, (d) BNNC@Top, (e) BNNC@Side systems

**Table S1**. The bond length and dipole moment of the most stable structures of BNNC@3SO_2_, BNNC@3SOF_2_, BNNC@3SO_2_F_2_, BNNC@Top, and BNNC@Side systems.

| Complex | Bond | Bond length(Å) | Dipole Moment |
| --- | --- | --- | --- |
| **BNNC@SO_2_** | R_S1-N1_ | 1.91 | 17.78 |
|  | R_O1-B2_ | 1.58 |  |
|  | R_O2-B1_ | 1.48 |  |
|  | R_O3-B3_ | 1.65 |  |
|  |  |  |  |
| **BNNC@SOF_2_** | R_S1-N1_ | 1.81 | 23.14 |
|  | R_F2-B1_ | 1.33 |  |
|  | R_F1-B2_ | 1.45 |  |
|  |  |  |  |
| **BNNC@SO_2_F_2_** | R_O1-B1_ | 1.53 | 23.35 |
|  | R_F1-B2_ | 1.69 |  |
|  |  |  |  |
| **BNNC@Top** | R_O1-B1_ | 1.44 | 19.64 |
|  |  |  |  |
| **BNNC@Side** | R_O1-B1_ | 1.41 | 19.42 |

**The XYZ coordination of optimized pristine BNNC with 180º disclination angle.**

90

B -1.43200 5.06830 -9.08820

N -0.65410 5.77910 -10.19780

N -2.89970 4.69050 -9.29970

B -1.43610 3.74840 -6.80290

N -0.66720 4.52160 -7.87820

N -2.82630 3.19130 -7.12400

B -3.53020 3.61190 -8.41640

B -4.88620 1.34670 -8.41330

N -4.68530 2.76420 -8.95740

N -5.54200 0.27780 -9.29150

B -1.43160 2.35790 -4.56650

N -0.67770 3.22620 -5.57860

N -2.63860 1.54890 -5.06150

B -3.31550 1.95020 -6.37400

B -4.02990 -0.55450 -6.78350

N -4.18550 0.93230 -7.11670

N -4.35240 -1.59810 -7.85430

B -5.18680 -1.19500 -9.07180

B -4.70410 -3.55180 -10.17420

N -5.46190 -2.22380 -10.16930

B -1.45350 0.85130 -2.44650

N -0.69430 1.87810 -3.30780

N -2.05430 -0.29490 -3.27650

B -2.80630 0.11330 -4.54740

B -2.46360 -2.29170 -5.55450

N -3.22970 -0.96580 -5.54600

N -2.51500 -3.17740 -6.80120

B -3.58610 -2.92370 -7.86360

B -2.56950 -4.88190 -9.32220

N -3.66590 -3.83870 -9.08760

B -0.01760 -0.91240 -1.12120

N -0.78930 0.44580 -1.12830

N -0.01070 -1.71290 -2.42810

B -1.29060 -1.63350 -3.27900

B 0.00170 -3.06890 -5.07280

N -1.31810 -2.47990 -4.55710

N 0.00770 -3.83300 -6.39920

B -1.30810 -4.05280 -7.15010

B 0.01880 -5.38280 -9.01200

N -1.30600 -4.85050 -8.45690

B 1.42980 0.85240 -2.43180

N 0.75240 0.44650 -1.12040

N 2.04110 -0.29320 -3.25480

B 1.27660 -1.63130 -3.26760

B 2.46970 -2.28500 -5.53410

N 1.31540 -2.47480 -4.54740

N 2.53080 -3.16620 -6.78360

B 1.32860 -4.04420 -7.14340

B 2.60150 -4.85600 -9.31580

N 1.33670 -4.83820 -8.45240

B 1.42700 2.35550 -4.55420

N 0.67810 1.87740 -3.30190

N 2.64220 1.55020 -5.03450

B 2.80850 0.11560 -4.51640

B 4.06050 -0.55130 -6.73660

N 3.24280 -0.96320 -5.51050

N 4.39170 -1.59370 -7.80630

B 3.61410 -2.91270 -7.83340

B 4.74910 -3.53160 -10.13910

N 3.69980 -3.81930 -9.06340

B 1.45300 3.74540 -6.79090

N 0.68330 3.22400 -5.57350

N 2.84640 3.18870 -7.09940

B 3.33260 1.95170 -6.34010

B 4.93080 1.34920 -8.35870

N 4.21760 0.93610 -7.06830

N 5.60760 0.28090 -9.22130

B 5.24580 -1.19120 -9.01030

N 5.52480 -2.21450 -10.11160

B 1.46990 5.06790 -9.07500

N 0.69440 4.52110 -7.87210

N 2.93730 4.68460 -9.27730

B 3.55860 3.60520 -8.38860

N 4.71780 2.75780 -8.92020

N 0.70210 5.77920 -10.19150

H 5.14800 3.00820 -9.78290

H -4.80500 -4.27110 -11.09240

H -2.58010 -5.51870 -10.30520

H 0.02350 -5.96790 -10.02690

H 2.61280 -5.47660 -10.30910

H 4.84410 -4.23820 -11.06780

H 6.10210 -1.96720 -10.88400

H 6.08380 0.55250 -10.05270

H 3.40340 4.95860 -10.11380

H 1.19840 6.14060 -10.97620

H -1.14280 6.14080 -10.98700

H -3.36010 4.96760 -10.13820

H -5.12050 3.02380 -9.81480

H -6.00400 0.54920 -10.13100

H -6.02840 -1.97720 -10.94980
